# Synergistic effects of cognitive impairment on physical disability in all-cause mortality among men aged 80 years and over: Results from longitudinal older veterans study

**DOI:** 10.1371/journal.pone.0181741

**Published:** 2017-07-26

**Authors:** Wan-Chen Yu, Ming-Yueh Chou, Li-Ning Peng, Yu-Te Lin, Chih-Kuang Liang, Liang-Kung Chen

**Affiliations:** 1 Center for Geriatrics and Gerontology, Kaohsiung Veterans General Hospital, Kaohsiung, Taiwan; 2 Aging and Health Research Center, National Yang Ming University, Taipei, Taiwan; 3 Center for Geriatrics and Gerontology, Taipei Veterans General Hospital, Taipei, Taiwan; 4 Division of Neurology, Department of Medicine, Kaohsiung Veterans General Hospital, Kaohsiung, Taiwan; 5 Institute of Environmental and Occupational Health Sciences, School of Medicine, National Yang-Ming University, Taipei, Taiwan; Istituto Di Ricerche Farmacologiche Mario Negri, ITALY

## Abstract

**Objective:**

We evaluated effects of the interrelationship between physical disability and cognitive impairment on long-term mortality of men aged 80 years and older living in a retirement community in Taiwan.

**Methods:**

This prospective cohort study enrolled older men aged 80 and older living in a Veterans Care Home. Those with confirmed diagnosis of dementia were excluded. All participants received comprehensive geriatric assessment, including sociodemographic data, Charlson’s Comorbidity Index (CCI), geriatric syndromes, activities of daily living (ADL) using the Barthel index and cognitive function using the Mini-Mental State Examination (MMSE). Subjects were categorized into normal cognitive function, mild cognitive deterioration, and moderate-to-severe cognitive impairment and were further stratified by physical disability status. Kaplan-Meier log-rank test was used for survival analysis. After adjusting for sociodemographic characteristics and geriatric syndromes, Cox proportional hazards model was constructed to examine associations between cognitive function, disability and increased mortality risk.

**Results:**

Among 305 male subjects aged 85.1 ± 4.1 years, 89 subjects died during follow-up (mean follow-up: 1.87 ± 0.90 years). Kaplan-Meier unadjusted analysis showed reduced survival probability associated with moderate-to-severe cognitive status and physical disability. Mortality risk increased significantly only for physically disabled subjects with simultaneous mild cognitive deterioration (adjusted HR 1.951, 95% CI 1.036–3.673, p = 0.038) or moderate-to-severe cognitive impairment (aHR 2.722, 95% CI 1.430–5.181, p = 0.002) after adjusting for age, BMI, education levels, smoking status, polypharmacy, visual and hearing impairment, urinary incontinence, fall history, depressive symptoms and CCI. Mortality risk was not increased among physically independent subjects with or without cognitive impairment, and physically disabled subjects with intact cognition.

**Conclusions:**

Physical disability is a major risk factor for all-cause mortality among men aged 80 years and older, and risk increased synergistically when cognitive impairment was present. Cognitive impairment alone without physical disability did not increase mortality risk in this population.

## Introduction

Population aging is associated with various challenges to the healthcare system, especially in Taiwan, one of the fastest aging countries in the world. The prevalence of cognitive impairment has increased along with advancing age, and it may double every 5 years [1,2]. Dementia and cognitive impairment have become a major public health challenge because of the associated extraordinary burden of care and health service utilization [3], longer hospital stays [4,5], and subsequent development of disability [6–8], as well as increased mortality [9–11]. The adverse impact of cognitive impairment on long-term mortality among older adults without dementia was also reported to be significant [12,13].

Similar to the results for cognitive impairment, physical disability was associated with functional decline [14,15], hospitalizations [16], institutionalization [17] and mortality among community-dwelling and hospitalized older adult patients [18–21]. Both physical disability and cognitive impairment were common conditions with adverse outcomes in later life. Although several previous studies demonstrated that both cognitive impairment and disability were independent of adverse impact on mortality [22–26], the attributable mortality risk of cognitive impairment may be explained by functional disability [27]. Most previous studies focused on independent associations between cognitive impairment or disability and mortality, however, effects of the interrelationship between physical disability and cognitive impairment on mortality remain unclear. Since cognitive impairment and physical disability often co-exist, synergistic effects on long-term mortality may result from coexisting physical disability and cognitive impairment in older adults. Therefore, this study aimed to evaluate the potential synergistic effects of physical disability and cognitive impairment among older old men living in a retirement community in Taiwan.

## Methods

### Study population

The Longitudinal Older Veterans (LOVE) study was a prospective cohort study that recruited residents living in the Veterans Care Homes (VCHs) in Taiwan to follow changes of their long-term health. All data collection in the LOVE study complied with the national policy of the Veterans Affairs Commission (VAC) for care planning in all VCHs. In Taiwan, VCHs are similar to assisted living or retirement communities in the United States, and they are only for veterans. Residents with needs for skilled nursing care are transferred to Veterans Nursing Homes; and older adults with a diagnosis of moderate to severe dementia are not permitted to live in these Veterans Homes because they have lost self-care ability. In the present study, data of all VCH residents aged 80 years and older living in the Gang-shan VCH were retrieved for analysis. Residents with the following conditions were also excluded: (1) unable to communicate with research staff, (2) unable to provide informed consent, and (3) terminal conditions such as malignant cancer or expected life expectancy shorter than 12 months. For all participants in the LOVE study, research nurses at the participating VCHs collected demographic data and performed comprehensive geriatric assessment (CGA) periodically. After the initial assessment in 2012, all subjects were followed for mortality status until the end of 2015. The mortality status of the participants was obtained based on the monthly reports of the VCHs. Around 518 beds were originally available in general, and around 400 residents are usually living in Gang-shan VCH. We enrolled 412 residents in 2012. Among these residents, 341 were aged 80 years and older, 29 had no baseline Mini-Mental State Examination (MMSE) scores, and 7 did not complete the entire CGA. Finally, data from the remaining 305 subjects were included for further analysis.

### Measurements

Demographic data, including age, education level, marital status, smoking status, and body mass index (BMI), were collected by trained research nurses at Gan-sa VCH. CGA was administered by the nurses, including the following: visual and hearing impairments (defined by reporting that the sensory impairment had affected activities of daily living [ADL]), polypharmacy (defined as currently using > 4 prescription drugs for over 3 months except for topical agents), history of falls and urinary incontinence in the past year, depressive symptoms (evaluated by the Chinese version of 15-item Geriatric Depression Scale [GDS-15], defined by GDS-15 > = 5) [28], and multimorbidity (evaluated using the Charlson’s Comorbidity Index, CCI) [29].

Cognitive function was evaluated using the Chinese version of the MMSE [30]. In the present study, MMSE scores for the study subjects were divided into three groups as previously described [2, 31, 32], i.e. normal cognitive function (normal cognition (NC) group: MMSE>23), mild cognitive deterioration (MCD) group: MMSE between 18–23), and moderate-to-severe cognitive impairment (MSC) group: MMSE <18).

ADL were assessed using the Barthel index (BI), for which the total score is between 0–100 [33]. In the present study, subjects with BI lower than 85 were defined as physically disabled [34,35]. Therefore, all included subjects were categorized into three groups based on their cognitive status and each entity was sub-divided into two subgroups based on their physical disability status.

### Statistical analysis

In this study, continuous variables are presented as mean ± standard deviation, and categorical variables are expressed as numbers and percentages. Comparisons of characteristics between subjects with three different cognitive statuses were done by ANOVA. Comparisons between subjects with and without physical disability were done by independent Student’s t-test. The Chi-square test was used for comparisons of categorical variables. Survival analysis was performed using Kaplan-Meier analysis (log-rank test). Cox proportional hazards regression model was used to evaluate mortality risk among subjects with different physiocognitive statuses after adjusting for other covariates. All statistical analyses were carried out using SPSS 20.0 (SPSS Inc., Chicago, IL, USA). A two-tailed p value of <0.05 was considered statistically significant. The Bonferroni method was used to adjust multiple comparisons and to change the significance threshold.

### Ethical considerations

The protocol of the present study was approved by the Institutional Review Board of Kaohsiung Veterans General Hospital. Written informed consent was waived by the IRB because: 1) subjects faced minimal risk only, 2) all data collection was part of the national VAC policy for care planning in all VCHs, 3) data analysis was performed primarily to improve quality of care for all VCH residents according to the VAC policy, and 4) all data were analyzed anonymously. After obtaining IRB approval, interviewers fully explained the study purpose to each participant and agreement to participate in the study was obtained verbally from all participants prior to data collection.

## Results

Overall, data of 305 residents (mean age: 85.1 ± 4.1 years, range 80–100 years, all males) were retrieved for analysis. The mean duration of follow-up for all residents was 3.15 ± 0.96 years, but the mean duration for only those who survived during the study period was about 3.68 ± 0.10 years. During the follow-up period, 89 (29.2%) participants died, and the mean survival time was 1.87 ± 0.90 years. Among the 89 deaths, 18 occurred in the first year, 30 in the second year, 28 in the third year, and 13 in the fourth year. Among all participants, 2.6% (8/305) were married and 18.7% (57/305) had received no formal education (Table 1). The prevalence of sensory impairment was high (visual impairment 83%, and hearing impairment 72.8%), as was polypharmacy (66.9%). Over one-third of all participants had urinary incontinence (37%), while about one-quarter of participants (26.6%) reported previous history of falls. The prevalence of residents with depressive symptoms was 33.1% among all participants.

**Table 1:** Comparisons of demographic characteristics of subjects according to cognitive status.

	Total subjects	MMSE <18	MMSE 18–23	MMSE >23	P value
	N = 305 % or mean±SD	N = 39 % or mean±SD	N = 101 % or mean±SD	N = 165 % or mean±SD	
Age	85.1±4.1	86.9±4.0	85.3±4.4	84.6±3.8	0.006
Educational level‡					< 0.001
No formal education	57 (18.7%)	17 (10.3%)	32 (31.7%)	8 (20.5%)	
Higher than Primary school	248 (81.3%)	148 (89.7%)	69 (68.3%)	31 (79.5%)	
Marital status					0.150
Married	8 (2.6%)	0 (0%)	1 (1.0%)	7 (4.2%)	
Single, Widows/Divorced	297 (96.6%)	39 (100.0%)	100 (99.0%)	158 (95.8%)	
Smoker	62 (20.3%)	9 (23.1%)	20 (19.8%)	33 (20.0%)	0.900
Body mass index					0.192
BMI < 18.5	23 (7.5%)	4 (10.3%)	9 (8.9%)	10 (6.1%)	
18.5 < = BMI < 25	101 (33.1%)	7 (17.9%)	38 (37.6%)	56 (33.9%)	
BMI > = 25	181 (59.3%)	28 (71.8%)	54 (53.5%)	99 (60.0%)	
Charlson’s comorbidity index	1.06 ± 1.43	1.31±1.20	0.99±1.20	1.05±1.60	0.492
Visual Impairment	254 (83.3%)	35 (89.7%)	83 (82.2%)	136 (82.4%)	0.510
Hearing Impairment	222 (72.8%)	32 (82.1%)	76 (75.2%)	114 (69.1%)	0.208
Polypharmacy	204 (66.9%)	29 (74.4%)	63 (62.45)	112 (67.9%)	0.371
Urine Incontinence	113 (37.0%)	18 (46.2%)	46 (45.5%)	49 (29.7%)	0.015
Fall History	81 (26.6%)	8 (20.5%)	29 (28.7%)	44 (26.7%)	0.615
Depressive symptoms	101 (33.1%)	24 (61.5%)	32 (31.7%)	45 (27.3%)	< 0.001
ADL	87.9±20.0	65.8±30.7	86.1±20.6	94.3±10.1	< 0.001

For cognitive function, subjects were classified according to MMSE scores as normal (NC group: MMSE >23, n = 165), mild cognitive deterioration (MCD group: MMSE 18–23, n = 101), and moderate-to-severe cognitive impairment (MSC group: MMSE 0–17, n = 39). The numbers of deaths were 43 in the NC group, 29 in the MCD group, and 17 in the MSC group. Table 1 also summarizes comparisons of demographic characteristics between different cognitive states, showing that marital status, current smoking, BMI, CCI, sensory impairments, polypharmacy, and history of falls were similar between groups. However, residents with poorer cognitive function were older and had higher percentages of urinary incontinence, depressive symptoms, and low mean BI scores. Table 2 shows comparisons of the demographic characteristics between residents with and without physical disability. Overall, 237 subjects (77.7%) were physically independent, i.e. BI ≥ 85 in this study. Compared to physically independent subjects, subjects with physical disability were significantly older, had higher CCI scores, and had greater incidence of polypharmacy, hearing impairment, depressive symptoms, urinary incontinence, history of falls and poor cognitive performance. The numbers of deaths were 58 for residents without physical disability and 31 for those with physical disability.

**Table 2:** Comparisons of demographic characteristics of subjects with or without disability.

	Total subjects	Disability	No disability	P value
	N = 305 % or mean±SD	N = 68 % or mean±SD	N = 237 % or mean±SD	
Age	85.1±4.1	86.3±4.4	84.8±4.0	0.009
Educational level				0.803
No formal education	57 (18.7%)	12 (17.6%)	45 (19.0%)	
Higher than Primary school	248 (81.3%)	56 (82.4%)	192 (81.0%)	
Marital status				0.206
Married	8 (2.6%)	0 (0%)	8 (3.4%)	
Single, Widows/Divorced	297 (96.6%)	68 (100.0%)	229 (96.6%)	
Smoker	62 (20.3%)	11 (16.2%)	51 (21.5%)	0.335
Body mass index				0.107
BMI < 18.5	23 (7.5%)	9 (13.2%)	14 (5.9%)	
18.5 < = BMI < 25	101 (33.1%)	19 (27.9%)	82 (34.6%)	
BMI > = 25	181 (59.3%)	40 (58.8%)	141 (59.5%)	
Charlson’s comorbidity index	1.06 ± 1.43	1.40±1.27	0.97±1.46	0.028
Visual Impairment	254 (83.3%)	61 (89.7%)	193 (81.4%)	0.107
Hearing Impairment	222 (72.8%)	60 (88.2%)	162 (68.4%)	0.001
Polypharmacy	204 (66.9%)	56 (82.4%)	148 (62.4%)	0.002
Urine Incontinence	113 (37.0%)	44 (64.7%)	69 (29.1%)	< 0.001
Fall History	81 (26.6%)	27 (39.7%)	54 (22.8%)	0.005
Depressive symptoms	101 (33.1%)	46 (67.6%)	55 (23.2%)	< 0.001
MMSE	22.9±4.7	19.7±5.3	23.8±4.2	< 0.001

**Fig 1:**
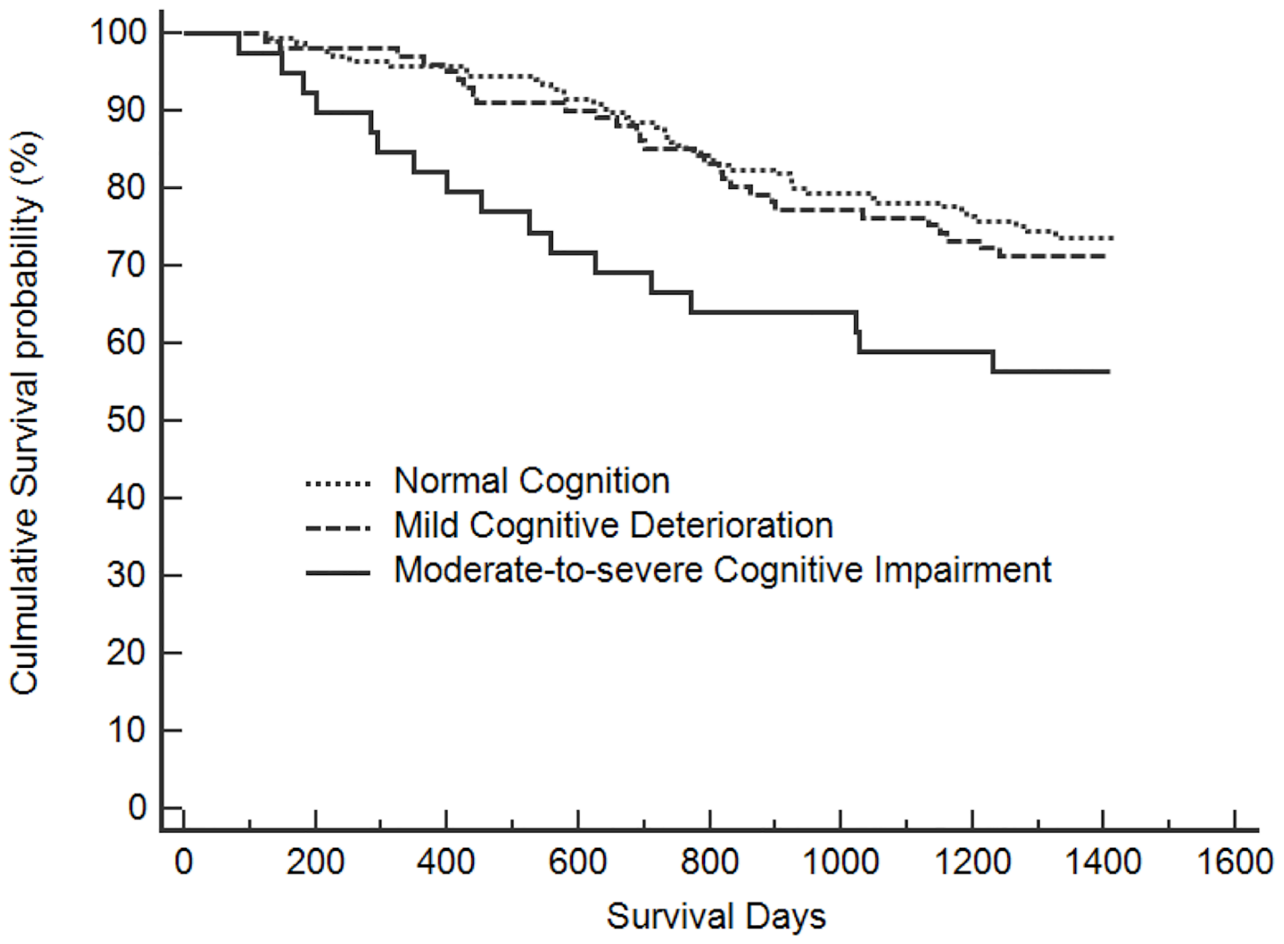
Kaplan-Meier unadjusted survival estimates according to cognitive status.

**Fig 2:**
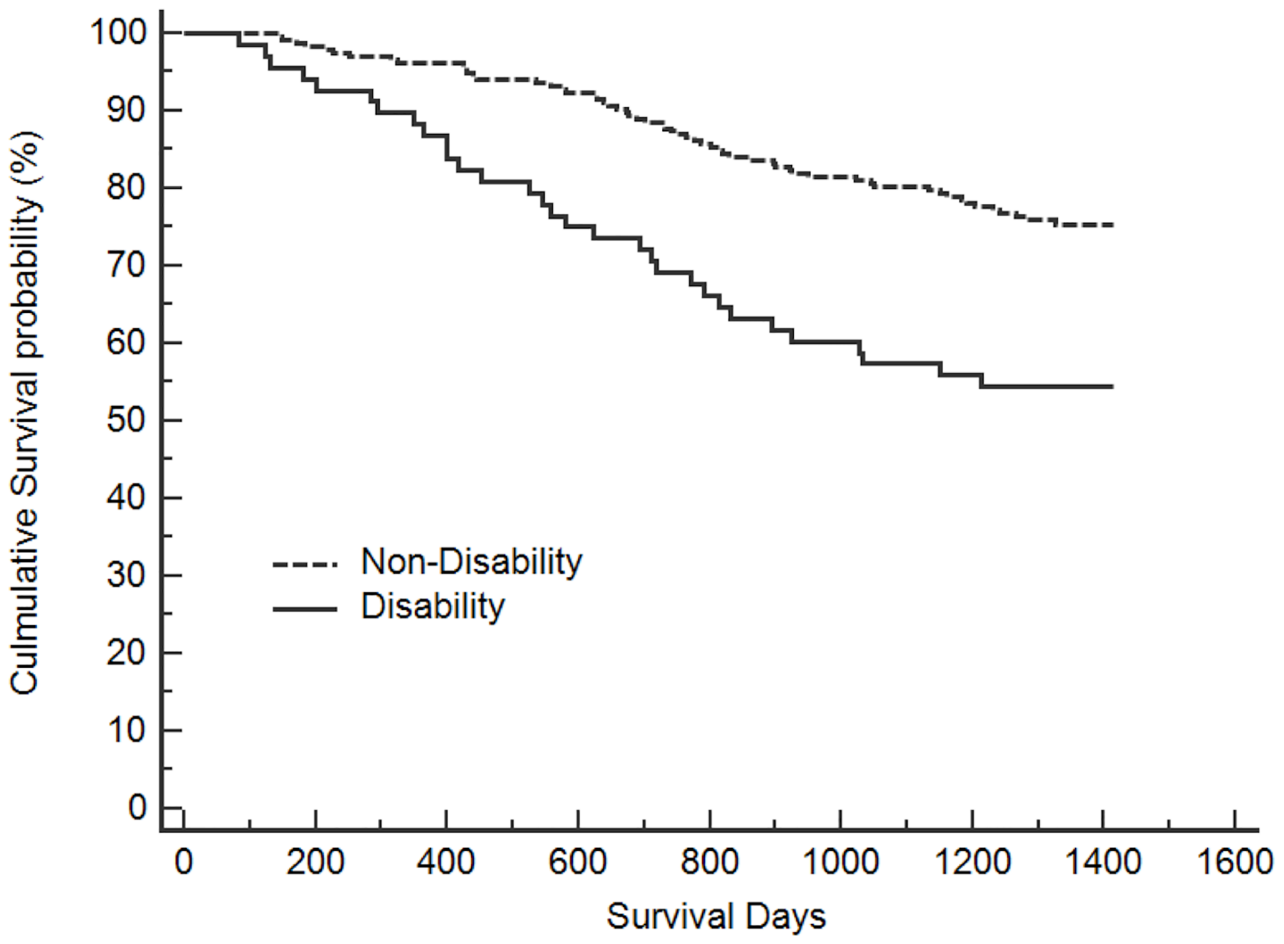
Kaplan-Meier unadjusted survival estimates according to disability status.

Kaplan-Meier analysis showed a reduction in survival probability associated with moderate-to-severe cognitive status and physical disability. Figs 1 and 2 show the results of survival analysis performed using Kaplan-Meier analysis (log-rank test). The subjects with NC and MCD had significantly lower mortality than those with MSC (groups for NC vs. MSC: log-rank test = 6.843, p = 0.009; groups for MCD vs. MSC: log-rank test = 3.962, p = 0.047). However, the difference in mortality between subjects with MCD and NC was not statistically significant (log-rank test = 0.250, p = 0.617). Significantly lower mortality was also found among those without physical disability compared to those with physical disability (log-rank test = 14.990, p < 0.001).

Associations between all-cause mortality and subjects with different cognitive status were also assessed by the Cox proportional hazards model. The crude (unadjusted) model showed that all-cause mortality was 2.084 times higher among residents with MSC than in those with NC (unadjusted hazards ratio [HR] 2.084, 95% confidence interval [CI]1.188–3.656, p = 0.010), but all-cause mortality was not significantly higher for those with MCD (unadjusted HR 1.126, 95% CI 0.703–1.803, p = 0.622) compared to those with NC. The mortality rates of those with physical disabilities were 2.314 times higher than those of residents without physical disabilities (unadjusted HR 2.314, 95% CI 1.494–3.582, p<0.001).

**Fig 3:**
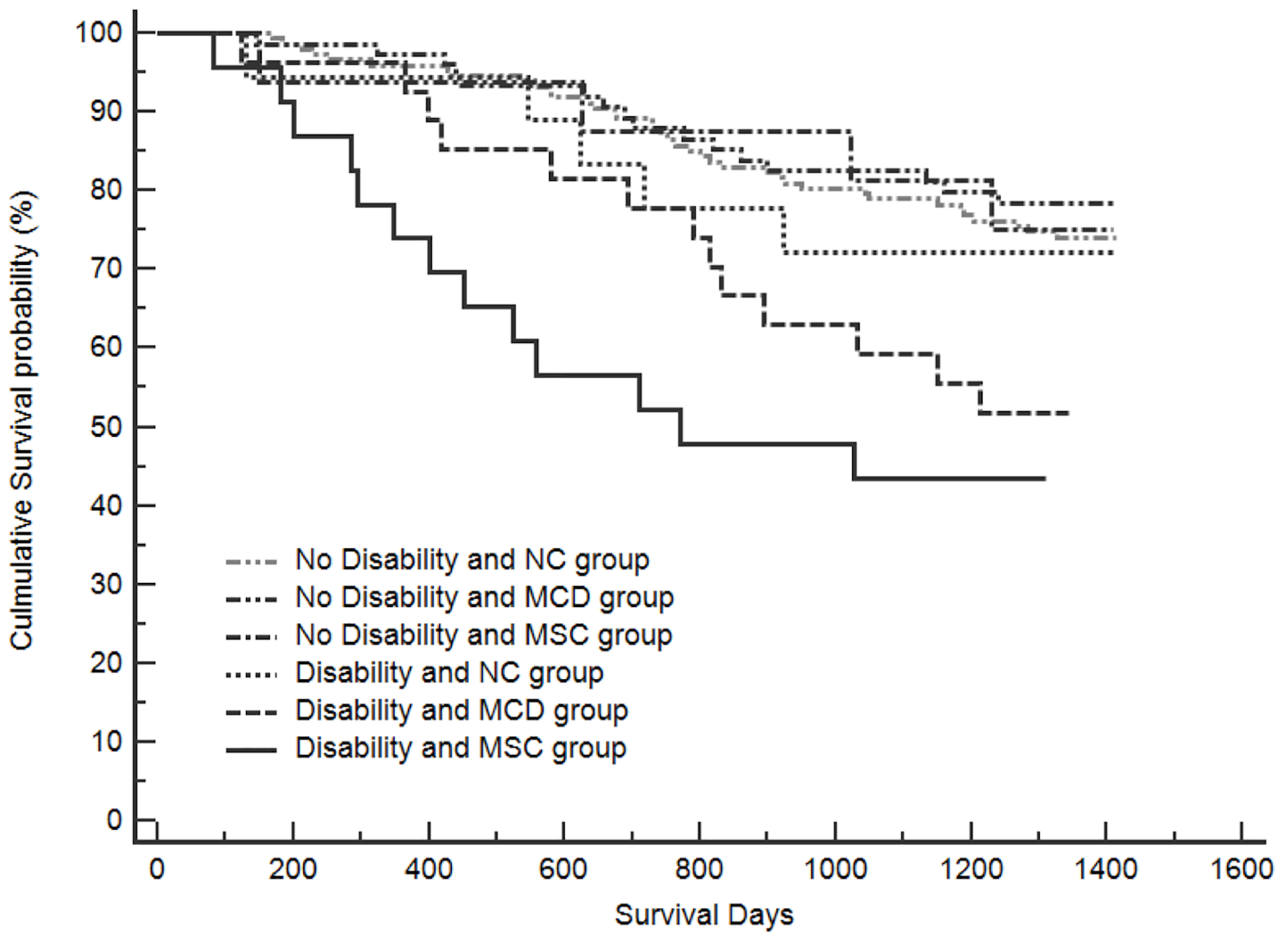
Kaplan-Meier unadjusted survival estimates according to combined function–cognition phenotypes.

As shown in Table 3, unadjusted and adjusted global p-values were initially analyzed for associations between the composite measure variables of MMSE and ADL and mortality using the Cox proportional hazards regression model (for MMSE, unadjusted HR 0.958, 95% CI 0.920–0.998, p = 0.042, and adjusted HR [aHR] 1.012, 95% CI 0.961–1.066, p = 0.650; for ADL, unadjusted HR 0.979, 95% CI 0.972–0.987, p<0.001, and aHR 0.984, 95% CI 0.973–0.994, p = 0.002). Additionally, we evaluated the synergistic effects of cognitive impairment and physical disability., Fig 3 shows the survival curves between the 6 groups based on the Kaplan-Meier unadjusted survival estimates. Compared to subjects without physical disability and NC, mortality risk was higher among the physically disabled subjects with MCD or MSC (unadjusted HR 2.179, 95% CI 1.160–4.094, p = 0.016 for disability & MCD group; unadjusted HR 3.355, 95% CI 1.784–6.312, p<0.001 for disability & MSC group). However, mortality risk was not significantly different between physically independent subjects with any cognitive status (NC, MCD, or MSC) and subjects with physical disability and intact cognition (NC). After adjusting for age, BMI, educational levels, smoking status, polypharmacy, visual and hearing impairment, urinary incontinence, history of falls, depressive symptoms and CCI, the mortality risk for groups of physically disabled subjects with MCD and MCS remained significant (aHR1.951, 95% CI 1.036–3.673, p = 0.038 for disability & MCD group; aHR2.722, 95% CI 1.430–5.181, p = 0.002 for disability & MSC group). Moreover, after correcting for multiplicity by the Bonferroni method, results remained significant for the group with physical disability and MSC.

**Table 3:** Association of combined function–cognition phenotypes with mortality among 305 elderly aged 80 and older living in Veterans Care Home.

	Model 1						Model 2					
	Unadjusted			Adjusted*			Unadjusted			Adjusted*		
Variables	HR	95% CI	p-value	HR	95% CI	p-value	HR	95% CI	p-value	HR	95% CI	p-value
Age	1.065	1.019–1.114	0.006	1.062	1.010–1.116	0.019	1.065	1.019–1.114	0.006	1.067	1.017–1.119	0.008
CCI	1.172	1.042–1.319	0.008	1.184	1.023–1.369	0.023	1.172	1.042–1.319	0.008	1.159	1.016–1.321	0.028
MMSE	0.958	0.920–0.998	0.042	1.012	0.961–1.066	0.650						
ADL	0.979	0.972–0.987	< 0.001	0.984	0.973–0.994	0.002						
Groups by the cognitive and physical function ^#^												
A: No disability & NC							Reference	Reference		Reference	Reference	
B: No disability & MCD							0.825	0.460–1.480	0.519	0.789	0.438–1.424	0.432
C: No disability & MSC							0.967	0.345–2.709	0.949	0.813	0.286–2.306	0.697
D: Disability & NC							1.149	0.452–2.919	0.771	1.019	0.400–2.597	0.968
E: Disability & MCD							2.179	1.160–4.094	0.016	1.951	1.036–3.673	0.038
F: Disability & MSC							3.355	1.784–6.312	< 0.001	2.722	1.430–5.181	0.002

Model 1: Analyze the unadjusted and the adjusted global HR, 95% CI and p-values of the composite measure by MMSE and ADL

Model 2: Analyze the unadjusted and the adjusted HR, 95% CI and p-values of the six classes made up by cognitive and physical status

* Adjusted for age, educational level, Smoker, Visual/Hearing impairment, BMI groups, CCI, polypharmacy, urine incontinence, fall history, Depressive symptoms based on GDS-15

*Abbreviations*: HR, hazard ratio; CI, confidence interval; ADL, Activity of daily living (Barthel index); MMSE, mini-mental state examination

^#^ Groups of cognitive function: NC: normal cognition; MCD: mild cognitive deterioration; MSC: moderate-to-severe cognitive impairment.

## Discussion

Both physical disability and cognitive impairment have been considered important risk factors associated with healthy aging, quality of life, and mortality among older adults, but these factors have usually been examined separately. However, during the aging process, physical disability and cognitive impairment often co-exist and closely interact, regardless of which came first. To the best of our knowledge, only a few studies have explored the interrelationship and prognostic impact of cognitive impairment and physical disability on long-term mortality among older adults. In the present study, the complex interrelationship between cognitive impairment, physical disability, and long-term mortality was examined among men aged 80 years and older. Physical function based on ADL scores and cognitive status based on MMSE scores were both associated with long-term mortality in univariate analysis. However, after applying the multivariate Cox proportional hazards model, participants’ ADL scores were independently associated with long-term mortality, but cognitive status scores were not. In addition, differences in mortality risk among physically independent participants were not significant between any cognitive statuses. For those with physical disability, the mortality risk increased only for physically disabled subjects with simultaneous mild cognitive deterioration or moderate-to-severe cognitive impairment, but not for those with intact cognition. Overall, the synergistic effects on long-term mortality occurred only for those with cognitive impairment who also demonstrated physical disability.

Previous studies have demonstrated that cognitive impairment is an independent predictor of mortality for adults aged 80 years and older after adjusting for many diverse covariates [12, 25, 26, 31, 32, 36, 37]. A 2-year longitudinal household follow-up study by Ramos et al. [25] enrolled 1667 urban community residents aged 65 years and older (including around 370 subjects aged 80 and older); after controlling for age, gender, geriatric syndrome (incontinence, falls, dysthymia), ADL, and other factors, but not chronic disease, mortality risk increased significantly for very old subjects (80 years vs. 65 years), extremely dependent subjects (7 vs. 0 ADLs), and those with severe cognitive impairment (<18 vs. 30 MMSE). Gustafson et al. [26] reported that global cognition (< = 24 on the MMSE) and physical disability were significant predictors for 7-year mortality in 668 long-lived Italian subjects aged 70 years or older (mean age 84±8 years) after adjusting for age, gender, BMI, APOEe4, ADL, and chronic diseases. In Sweden, an 8-year population-based prospective cohort study focused on non-dementia among people aged 75–95 years, concluding that cognitive impairment was a major risk factor for mortality, and its effect was not mediated by physical disability or other factors [12].

Two other reports, Takata et al. [36] and Nybo et al. [31], focused on very old populations (N = 207, all = 85 years old; N = 2262, > = 90 years old), and both demonstrated that poorer cognitive performance predicted mortality during 15-month and 10-year follow-ups. However, Takata et al. adjusted for covariates of gender, education, chronic disease, and diet only. In addition, Bruce et al. [37] followed middle-aged and elderly participants from the New Haven Epidemiologic Catchment Area Study for 9 years, finding that cognitive impairment had significant adverse effects after controlling for age, education, chronic conditions, disability, and depression, but the influence weakened with advanced age. In a longitudinal cohort study, Bassuk et al. [32] also followed residents in New Haven for 9+ years (with no overlap between participants in the study by Bruce et al.), demonstrating that mortality was more pronounced among younger respondents (< 80 vs. >80 years). Moreover, in that study, the severity of cognitive impairment based on baseline MMSE results was not independently associated with long-term mortality after adjusting for socio-demographic covariates, social behaviors, and health status. Only rapid decline from high normal cognition to severe cognitive impairment over a 3-year interval was associated with a significantly higher hazards ratio (3 times higher) in the following 0–6 years of follow-up for participants aged 80 years and older. Another study investigated the predictors of mortality in an aging community-based cohort, finding that the association between cognitive impairment (MMSE<24) and mortality disappeared after adjusting for IADL disability [24].

Our results did not show an independent association between MMSE scores and mortality when the model included ADL and other covariates such as age, education, smoking, BMI, polypharmacy, geriatric syndrome (visual/hearing impairment, fall history, urinary incontinence, and depressive symptoms). Although most studies only partly included subjects aged 80 years and older, Bruce et al. [37] and Bassuk et al. [32] both disclosed that the effects on long-term mortality were attenuated by age, especially for those aged 80 years and older. For several reasons, cognitive dysfunction predicted lower survival rates for older adults than for younger people. However, individuals of advanced age may report more incorrect responses for the MMSE due to poor memory, poor attention, hearing impairment, or physical dysfunction, which may change the values for differentiating between normal cognition and cognitive impairment. Another reason for lower cognitive function may include a different level of risk for mortality. For example, the residents with the same MMSE scores who belonged to the amnestic mild cognitive impairment group would be more likely to have diagnoses of Alzheimer disease in later years compared with the non-amnestic type [38]. In addition, residents with vascular dementia have higher risk of cardiovascular diseases, which could also interfere by influencing long-term mortality. The present study did not actually include the influence of long-term survival derived from cardiovascular or other individual diseases even though CCI was discussed. Impaired cognition is also associated with depression, delirium, clinical conditions, and risk for geriatric syndromes in very old adults, especially functional disability or frailty. These problems may attenuate the effect of cognitive impairment on longevity. As already mentioned, higher mortality due to rapid decline from normal cognition to severe cognitive impairment could cause higher mortality among those aged 80 years and older [32]. This rapid decline may be due to other problems rather than the cognitive impairment itself. Cano et al. [39] reported longitudinal analyses using data from the Hispanic Established Populations for the Epidemiologic Study of the Elderly (1995–96/2004–05) for Mexican Americans aged 67 and older. Those authors found that frailty was a stronger predictor of mortality for these subjects than cognitive impairment; however, when both cognitive impairment and frailty status were added to the model, the HR of dying for individuals with cognitive impairment was not statistically significant. The present study further adjusted for multimorbidities, physical function, and certain geriatric syndromes that are also related to higher mortality risk, demonstrating the adverse effect of physical disability rather than that of cognitive impairment on mortality.

Other studies on older populations found significant associations between functional disability and cognitive impairment and an increased risk of mortality, independent of several confounding variables [12, 25, 26, 31, 32, 37]. The relationship between cognitive impairment and disability is multifaceted. For the purpose of investigating the synergistic relationship of cognitive and physical dysfunction, we categorized the study subjects based on the status of their cognitive impairment and physical disability. We found differences in risk of all-cause mortality associated with cognitive function between those with and without physical disability. For those without physical disability, no significantly higher risk of mortality was found for those with cognitive impairment compared to residents with normal cognition. Only residents with both physical disability *and* mild cognitive deterioration or moderate-to-severe cognitive impairment had higher risk of mortality compared to those without physical disability and cognitive impairment. However, the adverse effect of mild cognitive deterioration disappeared after correcting for multiplicity using the Bonferroni method. Despite the result of Bonferroni correction, the synergistic effects of physical disability and cognitive impairment still existed, and physical disability affected the risk of mortality for older adults with cognitive impairment. In addition, we found no independently higher risk of mortality for those with physical disability but with normal cognition. These findings may have occurred because the residents with intact cognition could overcome the adverse effects caused by physical disability and decrease the risk of mortality. Otherwise, none of the subjects enrolled in the study were lost in follow-up because all subjects were living in a residence-like veterans home with well-designed care management and on-site medical support. Residents with mild cognitive deterioration and moderate-to-severe cognitive impairment living in such a facility may well maintain daily living longer and delay physical disability, and might have represented similar survival rates regardless of normal cognition or cognitive impairment.

Nevertheless, the present study still has several limitations. First, study results were only investigated in older men in a veterans home and may not be extrapolated to people living in different environments. Nevertheless, previous reports have focused mainly on communities, and few studies have investigated residents living in an institution, so our analysis adds to the knowledge base. Second, the cross-sectional design in analysis limited the possibility to explore the interval changes of physical and cognitive function and their association with all-cause mortality. Third, MMSE may not be the best assessment instrument for cognitive function among non-demented older old adults.

### Conclusion

In conclusion, physical disability based on ADL scores rather than cognitive impairment is a major risk factor for all-cause mortality among men aged 80 years and older, and the risk increases synergistically when cognitive impairment is present. Cognitive impairment alone, without physical disability, does not increase the mortality risk among older old men.

## Supporting information

S1 File Dataset. (SAV)
